# Reply to Onkenhout et al. Comment on “van Gemert et al. Child Abuse, Misdiagnosed by an Expertise Center—Part II—Misuse of Bayes’ Theorem. *Children* 2023, *10*, 843”

**DOI:** 10.3390/children11121430

**Published:** 2024-11-26

**Authors:** Martin J. C. van Gemert, Marianne Vlaming, Peter G. J. Nikkels, Peter J. van Koppen

**Affiliations:** 1 Department of Biomedical Engineering & Physics, Amsterdam University Medical Centers, Location AMC, University of Amsterdam, 1105 AZ Amsterdam, The Netherlands; 2 Private Practice, Criminal Psychology and Law, 6986 CL Amsterdam, The Netherlands; 3 Department of Pathology, Wilhelmina Children’s Hospital, University Medical Center, 3584 EA Utrecht, The Netherlands; p.g.j.nikkels@umcutrecht.nl; 4 Department of Criminal Law and Criminology, Faculty of Law, Vrije Universiteit Amsterdam, 1081 HV Amsterdam, The Netherlands; p.j.van.koppen@vu.nl

We thank the authors for their Comments [1] on our paper [2].

It is, however, unfortunate that the Comments are based on the importance of what they do themselves, how they do it, and what they do not do, rather than on the issues at hand. Also, for bruises and rib fractures not caused by physical abuse, we used the term “accidental”, which was changed to “accident” in the Comments and also used as if accidents developed. The Comments were therefore unclear.

## 1. Short Description of the Case (from [2])

A Dutch (mother)–Armenian (father) girl, who had an uncomplicated term birth, developed small bruises on varying body locations, but not on her chest, from the age of two weeks onwards. Her paternal grandmother, living in the USA, also bruised easily. At 2 months of age, the girl was referred to a *Landelijk Expertise Centrum Kindermishandeling: LECK* (*Dutch Expertise Center for Child Abuse*), located at a university medical center, where LECK pediatricians immediately, before consultation, suspected child abuse. The threat of child removal made the parents comply with the hospital’s child abuse investigations, where three healed, not dislocated, anterolateral, asymptomatic, left-side rib fractures were found. The LECK child abuse program includes the use of likelihood ratios, part of Bayesian statistics, to reach a conclusion; see Equation (1) below. LECK works anonymously, without seeing the patient.

They estimated a likelihood ratio of 10–100 and concluded that abuse was 10–100 times more likely than an accidental cause. A child protection worker from the Dutch center for domestic violence and child abuse (in Dutch: *Veilig Thuis*, or *Save at Home*; child abuse is reported to this organization in the Netherlands) reported suspicion of aggravated assault to the police, which triggered an 8 month placement in foster care, specifically in a secret home, for the girl and her 20-month-older brother. Resolving the case required four civil procedures and one criminal court procedure. The children returned home after the mother was diagnosed with hypermobility-type Ehlers-Danlos Syndrome (hEDS), and a pediatric and radiologic second opinion confirmed that the mother’s hEDS could have affected the girl’s bruises and that her rib fractures could well have been developed perinatally, while the observed widened metaphyses suggested that an underlying bone disease could well be possible.

The Bayesian statistics for our case were as follows:

Probability that Bruises/Rib Fractures are caused by Physical Abuse = Likelihood Ratio × Physical Abuse Probability(1)

For our case, the *Likelihood Ratio* is how much more often bruises and rib fractures occur when abuse takes place versus non-abuse causes, and the *Physical Abuse Probability* is approximately 0.0029 [1].

## 2. General Reply

Our case is a 2-month-old girl who had bruises on her body from the age of 2 weeks onwards, except on her chest. Both their general practitioner and the Well-Baby-Clinic doctor reported to LECK that there were no concerns about child abuse, given the good parent–child relationship, and the openness of the parents in taking pictures of the bruises and showing these openly to everybody. Also, the child’s paternal grandmother bruised easily. Yet, when the daughter met the LECK physician at 2 months of age, child abuse was immediately suspected, before the meeting, which triggered the LECK procedure for child abuse.

The authors describe that they gave at least a likelihood ratio to the advisee, which was estimated to be 10–100. However, the very small physical abuse probability of 0.0029 resulted in the total for Equation (1) being as small as (10–100) × 0.0029 = (0.029–0.29), or a virtually negligible probability that the bruises and rib fractures were caused by physical abuse. This problem questions both the examination and intervention in child protection by LECK.

We disagree with the statement in [1] that we made several mistakes in our paper [2].

In our analysis, therefore, the LECK/Veilig Thuis child abuse procedure should not have taken place. Their hypotheses were that every calculation in our paper [2] is incorrect and our three so-called implicit assumptions, given on page 4 by the authors [1], are mistakes.

## 3. Reply to the Points

### 3.1. Point 2.1

We described our case very carefully, so Risk 2 was not relevant for us.

### 3.2. Point 3.1

The LECK authors ([1], page 3), “*found every calculation in our paper incorrect*”. We stress that the three points the authors think that we made on page 4 are *incorrect*. That is, they wrongly accused us of assuming implicitly on page 4 that “—*all children in the population that have not been abused had an accident*; that “—*it is impossible for a physically abused child to have rib fractures*;” and that “—*there is no information in the case other than the medical findings considered in the likelihood ratio provided by LECK*;”. We hypothesized that the “*accident*”, in the 2nd point above, replaced our word “*accidental*”; see point 3.3 below for an explication.

In our paper we wrote ([2], top of page 7) that “—*the complexity of Bayesian statistics makes it obvious that (child abuse pediatrics) physicians, child protection employees and judges have little or no idea what Bayesian statistics are and what they do. Combined with the fact that the status of LECK made their statements uncritically accepted by all involved, allows LECK to predict whatever they want, even if nonsense, by lack of a controlling mechanism*.” This is obviously what happened in our case.

Further: “*In future cases, we suggest that consulting an expert statistician in clinical practice and also in legal court procedures, could prevent this unwanted situation, which likely decreases the number of unfounded abuse suspicion cases, including temporary or even permanent foster care placement*”.

Also: “*In child abuse cases where Bayesian statistics could play a role,—, it is obvious that the correct relation should be used rather than the misused version of LECK. Then, it would make no sense anymore to use Bayes’ theorem for physical child abuse assessments, which may suggest the obsoleteness of LECK’s approach and may give more hope for caregivers who might otherwise become suspected of physical child abuse*”.

### 3.3. Point 3.2

The severe conclusions of Prinsen [3] on the study of abuse by Alink and colleagues, e.g., [4], that their method was not usable for the general Dutch population, focusing on prevention of and intervention in child abuse, was the reason we chose the other papers for our physical abuse incidence calculations.

Nevertheless, the prevalence of types of physical abuse incidences are close, between 0.0009 and 0.0026 (our case [2]), and 0.0029 (the authors, [1]). In particular, because the number of English children over the age of 18 is approximately 14.4 × 10^4^, compared to 2.06 × 10^4^ for Sweden (from the Internet), combining England and Sweden gives a physical abuse incidence for our case of about 0.0025. In other words, the correct application of Bayes’ Theorem produces a very low abuse estimate for the bruises/rib fractures for LECK, about (10–100) × 0.0029 = (0.029–0.29), a virtually negligible incidence.

In the 2nd paragraph of [1] (page 6, Section 3.2, Point 4), it is stated that “*Equation (3) in part II of the paper series, the abuse probability should be divided by the probability of an “accident” to convert this number to prior odds, or P(PhysicalAbuse)*.” However, in Equations (7a) and (7b), this is exactly what we did. The authors have interchanged “*accidental*” and “*accident*”. So, we did not reason that “—*given the injuries a child must have been abused or had an accident, as the authors assumed*”.

### 3.4. Point 3.3

Equation (3) of the authors is wrong; “*accident*” must be “*accidental*”, implying “*non-abuse*” or “*coincidental*”. Then, the authors’ comments on our derivation of the likelihood ratios are not very important, in view of the low physical abuse factors we and the authors did find (see above in our reply to point 3.2).

Further, the authors claim that “*Additionally, rib fractures as a result of childbirth are rarely seen*”, referring to three papers published in 1964, 1994, and 2009. We recently published a paper on physical abuse-related rib fractures in infants [5], which, in the Netherlands, had a 250 times *lower* incidence than the sum of all the non-abuse-related rib fractures that we included (from: birth trauma, prematurity, osteogenesis imperfecta, hEDS, severe chronic placental pathology, and vitamin-D deficiency).

### 3.5. Point 3.4

The likelihood ratio was guesstimated to be 10–100, which implies that, given LECK’s status, their statements were uncritically accepted, allowing LECK to predict whatever they want, even if nonsense.

In the Discussion of [2], we stated (page 5): “*In Part I, we explained that LECK offers healthcare professionals an anonymous advice without requesting permission from caregivers and without observing the child themselves*.” (underlining by us).

The remaining paragraph, with statements numbered 1, 2, and 3, is scientifically unfounded for reply.

### 3.6. Point 4 Conclusions

Given the above replies, we disagree with the statement in [1] that we made several mistakes in our paper [2].

## 4. Our Conclusions

In conclusion, LECK should be aware of two things. First, their use of Bayes’ Theorem, but only the likelihood ratio, and, second, their analysis of patients they do not know and do not observe, will still be uncritically accepted without any problem by virtually everybody. A likelihood ratio of 10–100, implying that abuse is a more likely cause than other accidental causes, will be uncritically accepted too. For us, however, the full Bayes’ Theorem should be used, which implies a negligibly small physical child abuse probability, as the likelihood of physical abuse causing the symptoms (bruises and rib fractures) is so small; see also point 3.2. Therefore, it does not make sense to use Bayes’ Theorem for child abuse assessments, which may provide more hope to caregivers who might otherwise be wrongly suspected of having committed physical child abuse.

Thus [6], to prevent cases like this from happening again, LECK should stop using Bayesian statistics, especially invalid likelihood ratios, stop using its anonymous methodology, and increase its knowledge of rare and metabolic diseases that may present as symptoms that could mimic child abuse. The parents should be thoroughly investigated by an independent experienced pediatrician, together with an experienced pediatric clinical psychologist to produce an independent opinion. Children deserve this extra safeguard before being separated from their parents.
